# Supplementary figures and images for: Loss of Tid1/DNAJA3 Co-Chaperone Promotes Progression and Recurrence of Hepatocellular Carcinoma after Surgical Resection: A Novel Model to Stratify Risk of Recurrence
Source: Cancers (Basel). 2021 Jan 4;13(1):138. doi: 10.3390/cancers13010138 (PMC7795123; doi:10.3390/cancers13010138)

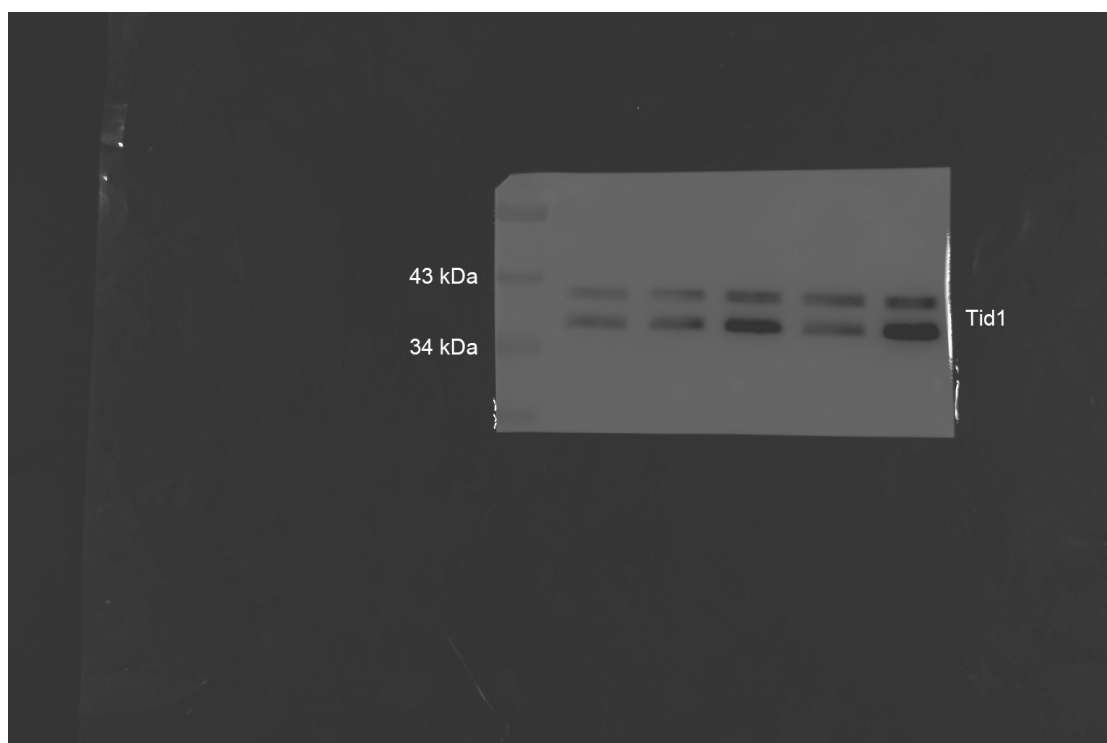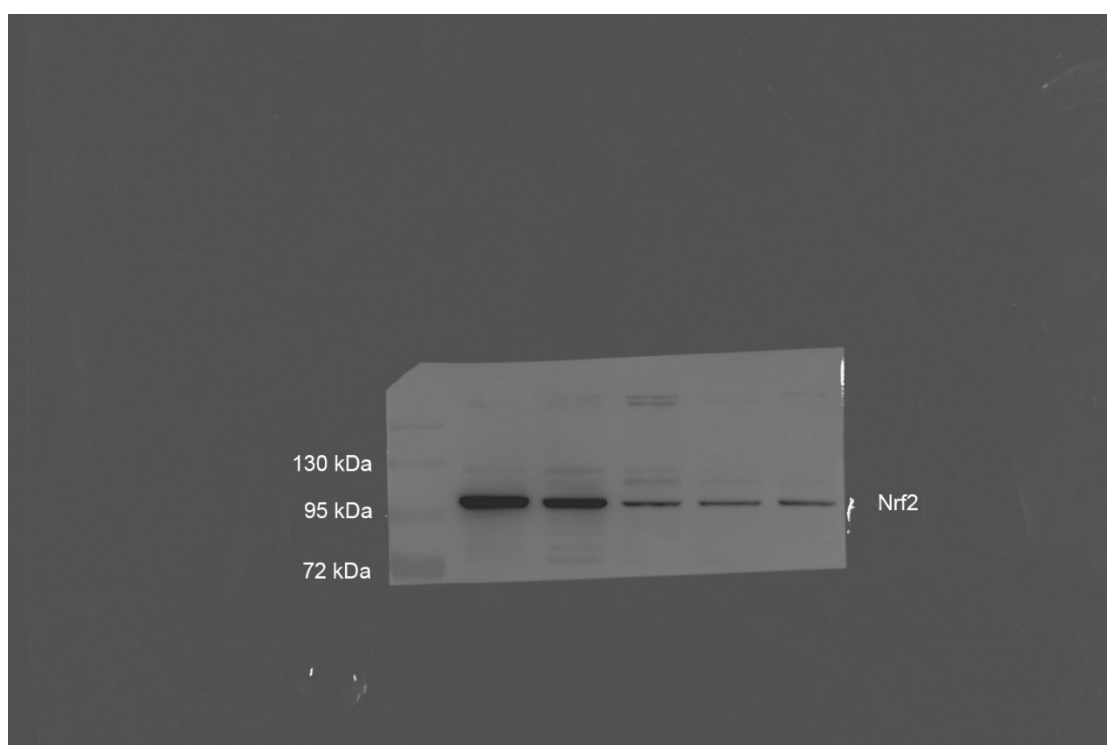

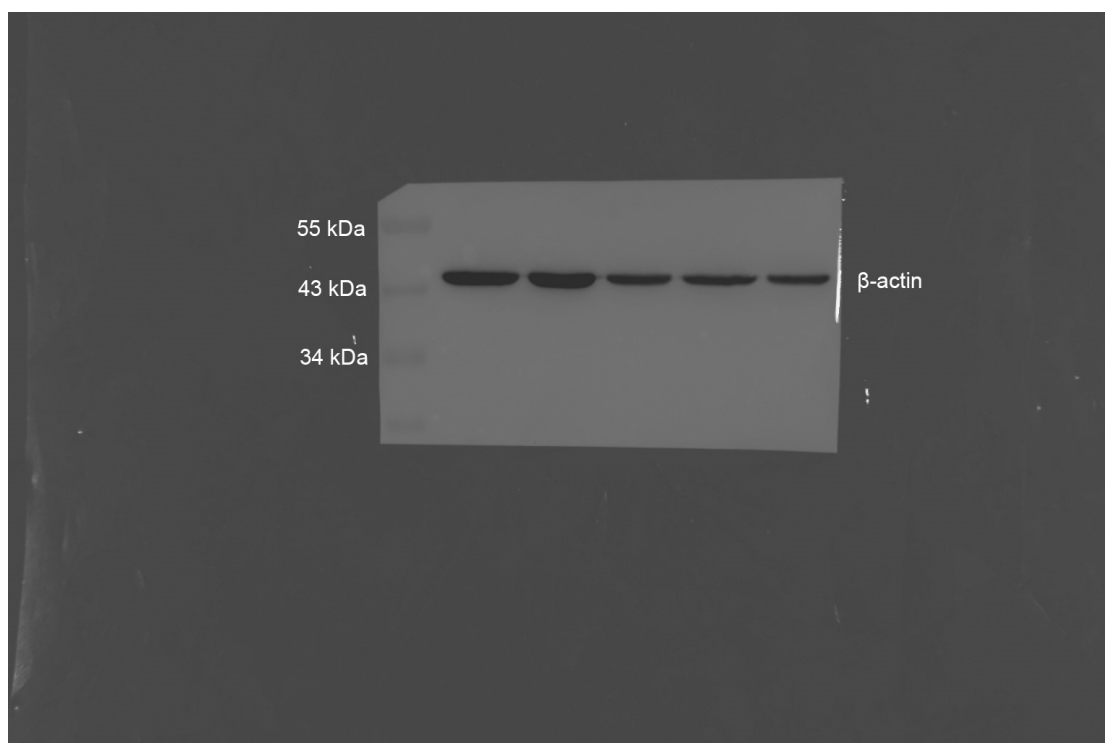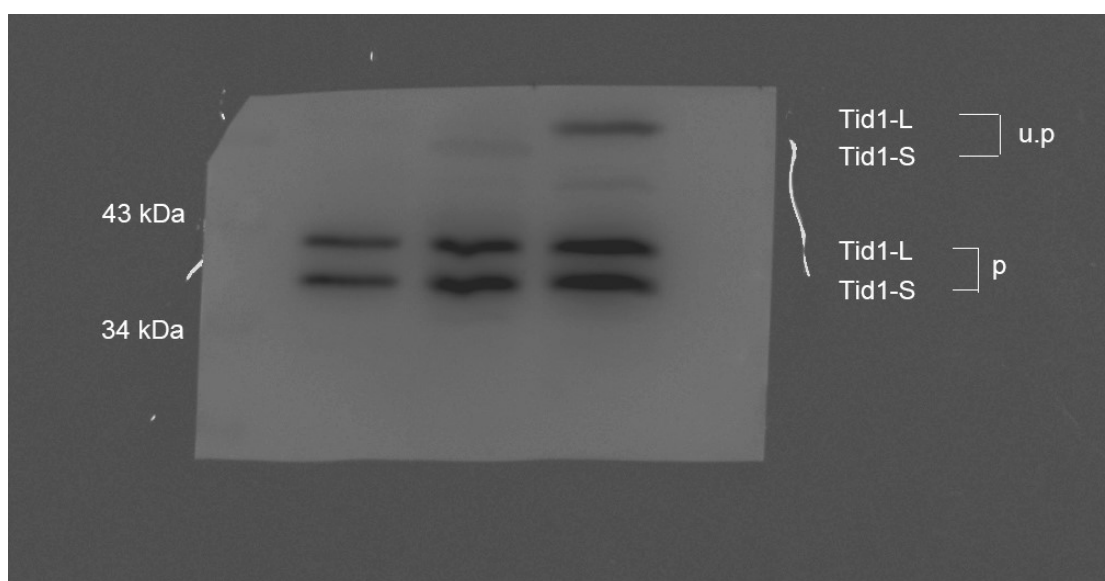

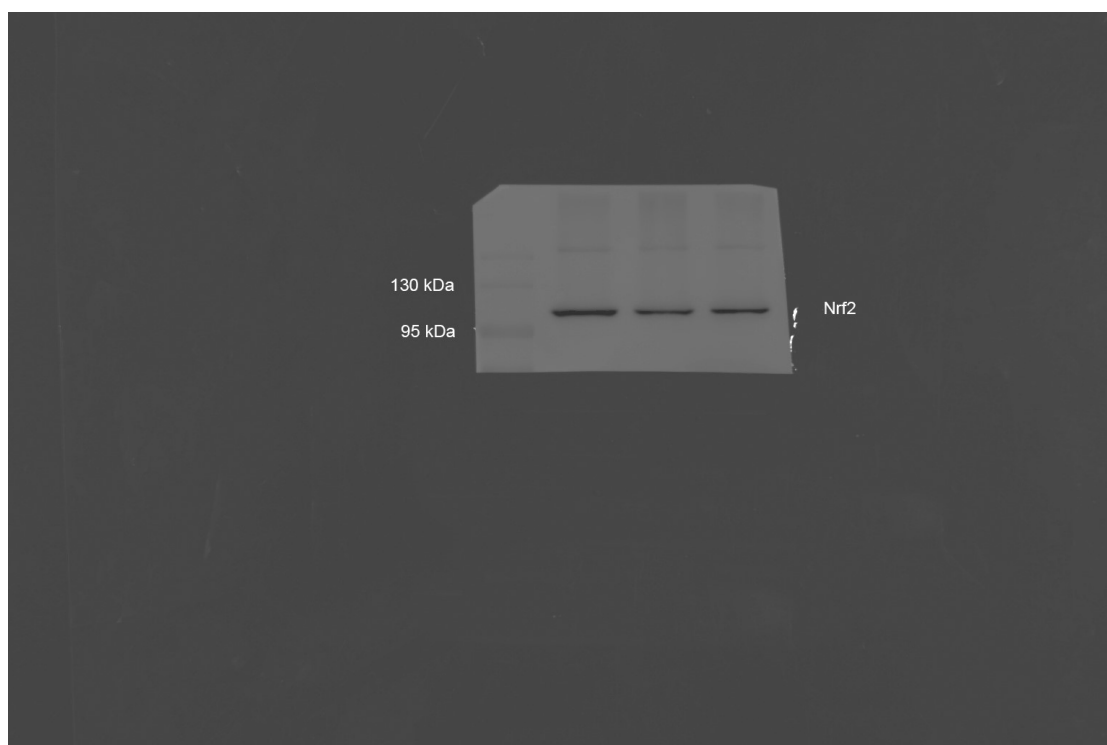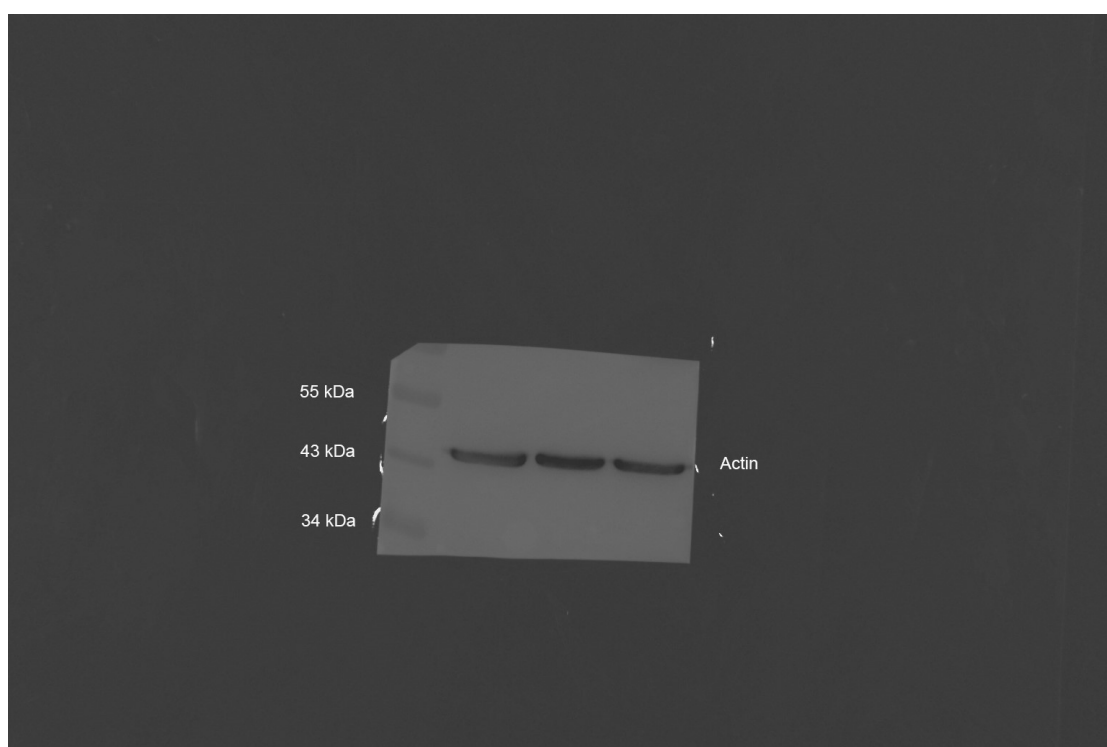

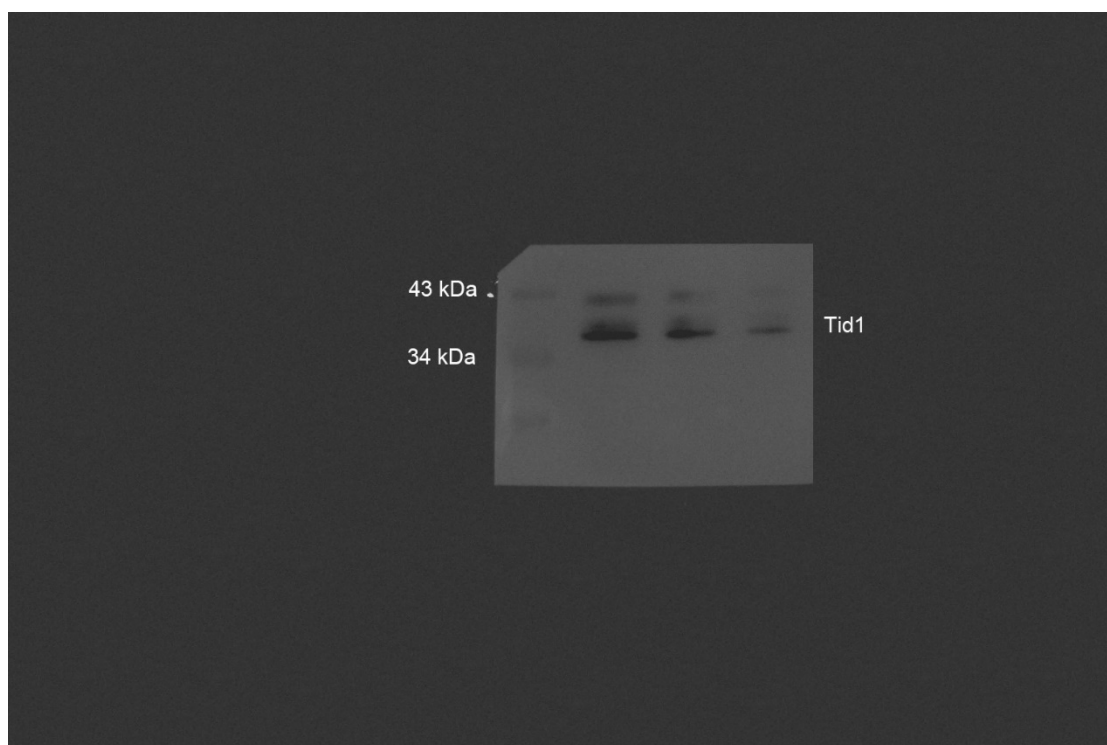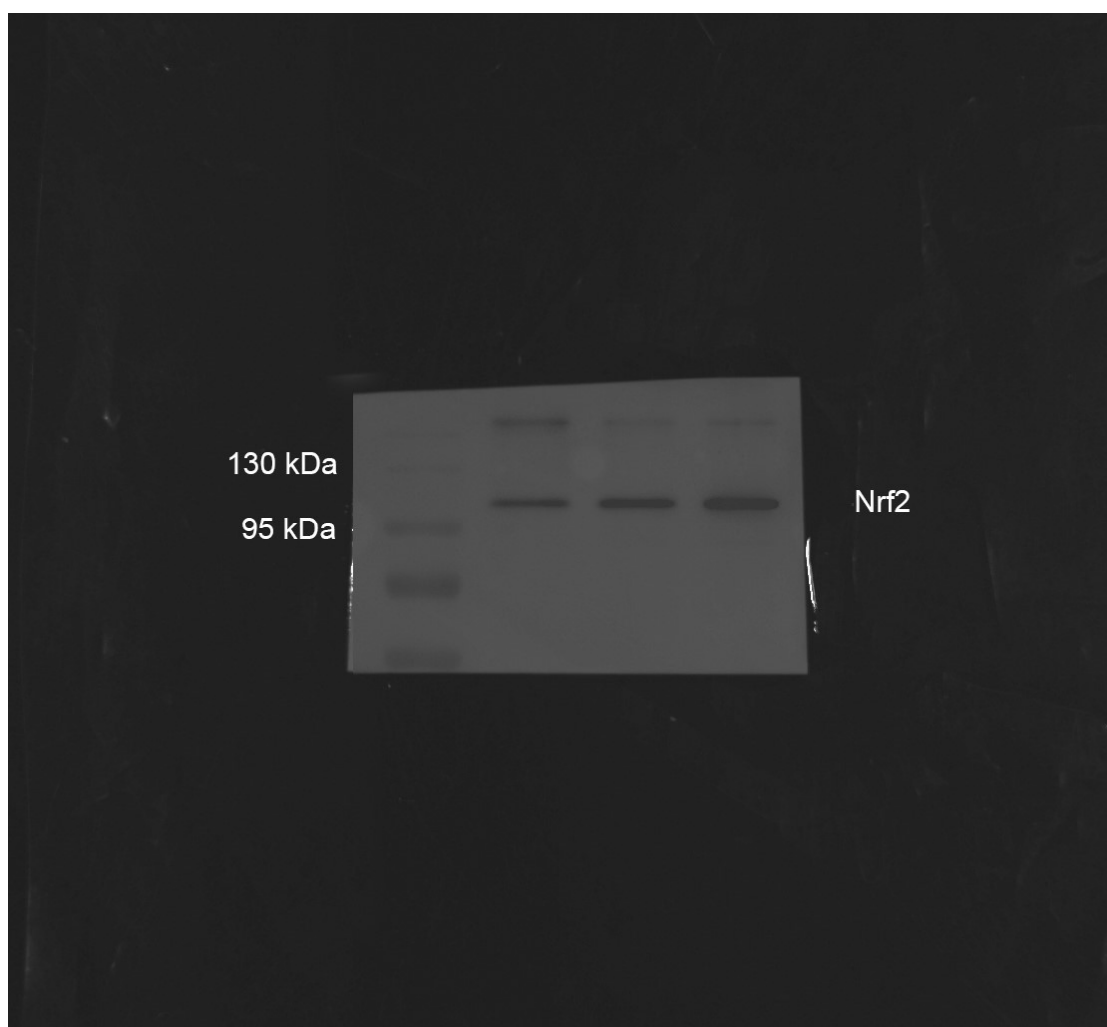

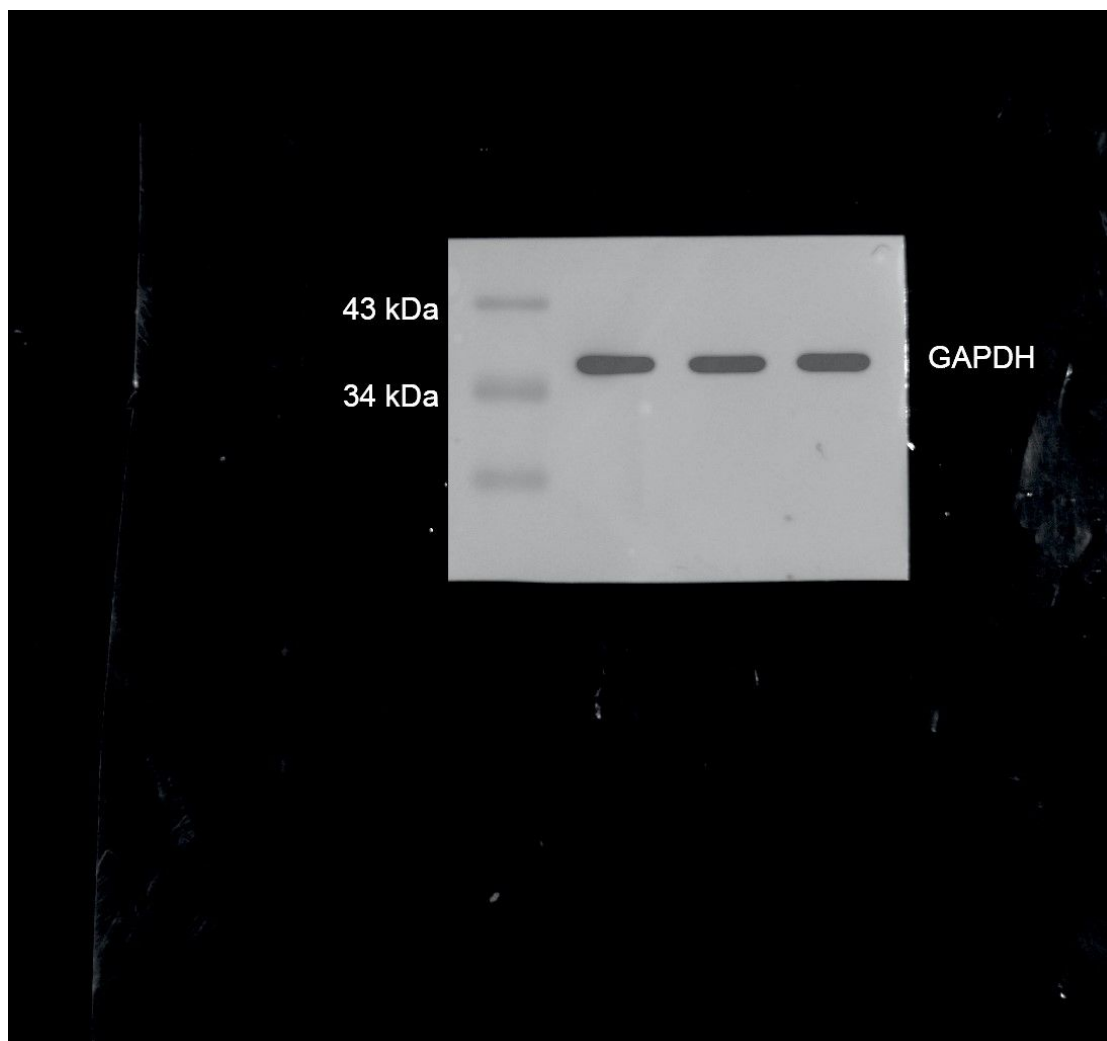

Supplement: Supplementary file 1 [file cancers-13-00138-s001.pdf]
